# A Fast and Accurate Method to Identify and Quantify Enzymes in Brush-Border Membranes: In Situ Hydrolysis Followed by Nano LC-MS/MS

**DOI:** 10.3390/mps3010015

**Published:** 2020-02-10

**Authors:** Antonio Brun, Melisa E. Magallanes, Carlos Martínez del Rio, Gregory A. Barrett-Wilt, William H. Karasov, Enrique Caviedes-Vidal

**Affiliations:** 1 Instituto Multidisciplinario de Investigaciones Biológicas de San Luis (IMIBIO-SL), Consejo Nacional de Investigaciones Científicas y Técnicas (CONICET), 5700 San Luis, Argentina; bruntonio@gmail.com (A.B.); melisa.eliana.magallanes@gmail.com (M.E.M.); 2 Facultad de Ciencias de la Salud. Universidad Nacional de San Luis, 5700 San Luis, Argentina; 3 Department of Zoology and Physiology, University of Wyoming, Laramie, WY 82071, USA; CmDelRio@uwyo.edu; 4 Mass Spectrometry and Proteomics Facility, Biotechnology Center, University of Wisconsin-Madison, WI 53706, USA; barrettwilt@wisc.edu (G.A.B.-W.); wkarasov@wisc.edu (W.H.K.); 5 Department of Forest and Wildlife Ecology, University of Wisconsin-Madison, Madison, WI 53706, USA; 6 Departamento de Biología, Universidad Nacional de San Luis (UNSL), 5700 San Luis, Argentina

**Keywords:** α-glucosidase, polyacrylamide gels, detection, proteomics, activity

## Abstract

A simple method for the identification of brush-border membrane α-glucosidases is described. The proteins were first solubilized and separated in a gel under native, non-denaturing, conditions. The gel was then incubated in substrate solutions (maltose or sucrose), and the product (glucose) exposed in situ by the oxidation of o-dianisidine, which yields a brown-orange color. Nano-liquid chromatography coupled to mass spectrometry analyses of proteins (nano LC-MS/MS) present in the colored bands excised from the gels, was used to confirm the presence of the enzymes. The stain is inexpensive and the procedure permits testing several substrates in the same gel. Once enzymes are identified, their abundance, relative to that of other proteins in the brush border, can be semi-quantified using nano LC-MS/MS.

## 1. Introduction

It is a common goal in physiology to assess the function of enzymes as well as to identify effector proteins in subcellular compartments. Numerous methods have been proposed based on split assays, one to measure the function of the enzyme and another to identify the protein that catalyzes the reaction. The latter step often requires an additional sample (not the one in which function was measured), and relies on the use of antibodies. However, antibodies typically are species-specific and do not cross-react when studying non-traditional model animals, or are not specific enough to distinguish between closely related enzymes. Specific antibodies can require time to be produced. We describe a method with the following two advantages: (1) It can perform both steps on the same sample, and (2) the enzyme identification is fast and accurate. We used the method to reveal the hydrolytic activity of α-glucosidases in brush border membrane (bbm) of intestinal epithelial cells (enterocytes), and their identification, by combining a gel electrophoresis for sample protein separation, a colored reaction to reveal the enzyme activity on the substrate, i.e., zymography [1], and a proteomic method, for enzyme identification-nano-liquid chromatography coupled to mass spectrometry (nano LC-MS/MS ) of the solubilized stained bands of the gels. If the method is followed by nano LC-MS/MS on the proteins from the bbm, the method also allows quantifying the relative abundance of the enzymes identified.

Our demonstration target enzymes hydrolyze the final products of the degradation of starch and glycogen (e.g., maltose and maltoligosaccharides) and disaccharides (e.g., maltose, sucrose, isomaltose). In mammals, the intestinal α-glucosidases are the complexes sucrase-isomaltase (SI) and maltase-glucoamylase (MGAM) of the bbm of enterocytes. SI and MGAM are orthologs that derive from an ancient duplication and are similar in structure and composition, sharing a 60% homology between complexes [2,3]. Both enzyme complexes include two subunits, an *N*-terminal subunit (*N*t-MGAM and *N*t-SI) and a *C*-terminal subunit (*C*t-MGAM and *C*t-SI) [4,5]. According to the classification system of the carbohydrate-active enzymes (CAZy, [6]), all of the subunits belong to the glycoside hydrolase 31 family (GH31) subgroup 1, characterized by the WIDMNE sequence in their catalytic centers [7]. All these similarities make it time consuming and costly to distinguish the different complexes. Moreover, because some of the subunits share substrate hydrolytic specificity [8,9], and in the reaction assay the activity gives similar colored products, distinguishing between the enzymes poses an even greater challenge. Therefore, we adapted a method using zymography to separate and detect activities plus nano LC-MS/MS to recognize the proteins in the gels slices where the reaction was apparent. We used a preparation of bbm of the small intestine of laboratory mice and two substrates hydrolyzed by the complexes, maltose and sucrose. According to the specificities of these enzymes, the proteomic analyses of the gel slices should reveal the presence of both proteins.

## 2. Material and Methods

### 2.1. Brush Border Membrane Preparation and Zymography

Dr. Sarah Newman from the University of Wisconsin—Madison Research Animal Resource Center (RARC) provided the study animals. The gastrointestinal tracts of two Swiss-Webster mice anesthetized with isoflurane were removed. The small intestine from the pyloric valve to the ileocecal junction was excised and placed in ice cold PBS solution for enterocyte isolation. Immediately after, animals were euthanized by anesthesia overdose. The isolation of enterocytes and bbm were performed according to protocols described by Mac Donal, et al. [10] and McConnell, et al. [11]. 

**Figure 1:**
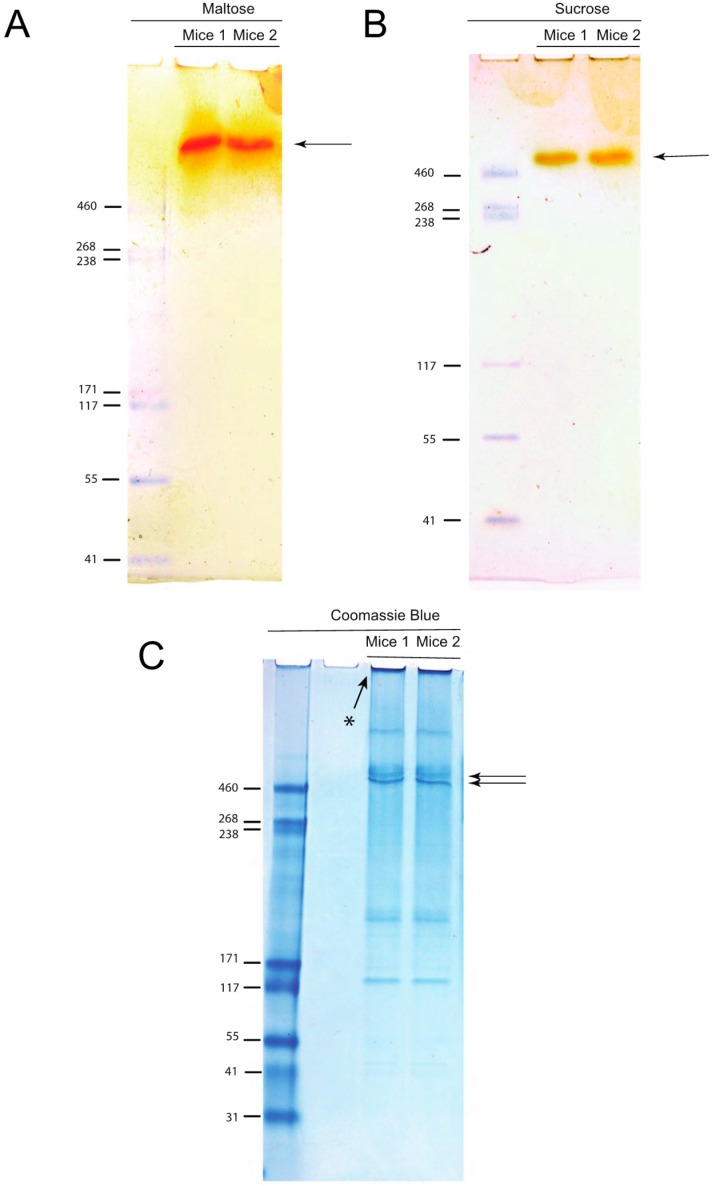
Bands developed indicating α-glucosidase activity by zymography. The polyacrylamide gels were incubated with either maltose (**A**) or sucrose (**B**) solution or Coomassie blue solution (**C**). Then for A and B only, the gel was immersed in assay reagent solution until a well-defined band appeared, as shown. Asterisk (*) on Figure 1C shows proteins that did not run through the gel.

Proteins from the bbm preparations were solubilized in loading buffer (60 mM Tris-HCl pH 6.8, 20% Glycerol, 1% Triton X 100 and 0.01% Bromophenol blue) and then incubated 10 min at 40 °C in a water bath, vortexed, and 10 µg of total protein was loaded per gel well. For each mouse, three gels were run; one to reveal maltose hydrolytic activity (identified as I in Table 1), one for sucrose activity (identified as II in Table 1), and the remaining gel was used to visualize the proteins by Coomassie Blue staining following Simpson [12]. All the gels were run with PageRuler Plus Prestained Protein ladder (ThermoFisher Scientific catalog number 26,619, Waltham, MA, USA) as a molecular marker. Gels I and II were run at room temperature in 4–12% Tris-Glycine Mini polyacrylamide gel (Novex^TM^ TermoFisher Scientific) at 100 V constant and ~350 mA using Tris-glycine pH 8.3 as running buffer (Table 1). Coomassie blue gel running time was 2 h. Gels for disaccharidase activity were run for a total of 3 h to have better separation between these enzymes that have similar molecular weight and overlapping activity [8,9,13,14,15]. The two gels for disaccharidase activity were immersed in either maltose or sucrose 56 mM in maleate-OH buffer for 1 hour at 37 °C (Table 1). The buffered substrate solution was removed. Then the gels were soaked in assay reagent solution of the Glucose (GO) Assay Kit (Sigma-Aldrich GAGO20, Saint Louis, MO, USA) at 37 °C; for 1 h for the gel previously soaked in maltose, and for 5 h for the gel previously soaked in sucrose (Table 1, Figure 1). The reaction is based on the promotion of the oxidation of glucose produced by the hydrolytic enzymes tested by glucose oxidase and formation of H_2_O_2_ by a peroxidase, which in turn oxidizes the colorless-reduced o-dianisidine forming an orangish compound according to the manufacturer (Glucose (GO) Assay Kit, Sigma-Aldrich GAGO20). The reaction was stopped by rinsing the gel with deionized (DI) water 3 times. Finally, the bands were cut and submerged in DI water and stored at 4 °C for further analysis. 

### 2.2. Enzymatic “In Gel” Digestion 

Stained gel slices from the native gel were excised based on the activity staining. Slices were placed for 5 min in MeOH/H_2_O/NH_4_HCO_3_ [50%:50%:25 mM], and the proteins entrapped in the gel were denatured for 10 min in SDS/DTT/Tris-HCl solution [2%/1 mM/50 mM pH 7] with subsequent 2 × 5 min washes in MeOH/H_2_O/NH_4_HCO_3_ [50%:50%:25 mM]. Gel fragments were washed twice for 5 min in MeOH/H_2_O/NH_4_HCO_3_ [50%:50%:100 mM], dehydrated for 5 min in acetonitrile (ACN)/H_2_O/NH_4_HCO_3_ [50%:50%:25 mM] then once more for 1 min in 100% ACN, dried in a vacuum concentrator (Speed-Vac, ThermoFisher Scientific) for 2 min, reduced in 25 mM DTT (dithiotreitol in 25 mM NH_4_HCO_3_) for 30 min at 52 °C, alkylated with 55 mM IAA (iodoacetamide in 25 mM NH_4_HCO_3_) in darkness at room temperature for 30 min, washed twice in H_2_O for 30 s equilibrated in 25 mM NH_4_HCO_3_ for 1 min, dehydrated for 5 min in ACN/H_2_O/NH_4_HCO_3_ [50%:50%:25 mM] then once more for 30 s in 100% ACN, dried again and rehydrated with 20 μL of trypsin solution [10 ng/μL trypsin (Promega, Madison, WI, USA) in 25 mM NH_4_HCO_3_/0.01% Protease MAX *w*/*v* (Promega)]. An additional 30 μL of digestion solution (25 mM NH_4_HCO_3_/0.01% Protease MAX *w*/*v*) was added to facilitate complete rehydration. The digestion was conducted for 3 h at 42 °C. The supernatant was removed, and gel pieces were extracted for peptides with 2 × gel volume of ACN/H_2_O/TFA solution [70/30/0.75%]. Extracted peptides were combined with the supernatant. Degraded Protease MAX was removed via centrifugation [max speed 16,000 g, 10 min] and the peptides solid phase extracted (ZipTipC18pipette tips Millipore, Burlington, VT, USA). The extracted peptides were subsequently submitted for nano-LC-MS/MS analysis. This protocol can be found at: https://www.biotech.wisc.edu/services/massspec/protocols/ingel.

### 2.3. Nano LC-MS/MS

Peptides were analyzed by nano LC-MS/MS using the Agilent 1100 nanoflow system (Agilent Technologies, Santa Clara, CA, USA) connected to a hybrid linear ion trap-Orbitrap mass spectrometer (LTQ-Orbitrap Elite™, ThermoFisher Scientific) equipped with an EASY-Spray™ electrospray source. Chromatography of peptides prior to mass spectral analysis was accomplished using capillary column with integrated emitter (PepMap^®^ C18, 3 µM, 100 A, 150 × 0.075 mm, ThermoFisher Scientific) onto which 3 µL of extracted peptides was automatically loaded. The nanoHPLC system delivered solvents A: 0.1% (*v*/*v*) formic acid, and B: 99.9% (*v*/*v*) acetonitrile, 0.1% (*v*/*v*) formic acid at 0.50 µL/min to load the peptides (over a 30 min period) and 0.3 µL/min to elute peptides directly into the electrospray source using a gradual gradient from 3% (*v*/*v*) B to 30% (*v*/*v*) B over 77 min and concluded with 5 min fast gradient from 30% (*v*/*v*) B to 50% (*v*/*v*) B at which time a 5 min flush-out from 50–95% (*v*/*v*) B took place. As peptides eluted from the HPLC-column/electrospray source survey, MS1 scans were acquired in the Orbitrap over the mass range 300 to 2000 *m*/*z* at resolving power of 120,000 followed by MS2 fragmentation in the linear ion trap of the 20 most intense peptides detected in the MS1 scan. Precursor redundancy was limited by dynamic exclusion.

## 3. Data Analysis and Protein Identification

Raw MS/MS data were converted to mgf file format using MSConvert (ProteoWizard: Open Source Software for Rapid Proteomics Tools Development). The resulting mgf files were used to search against the *M. musculus* (87,156 entries) Uniprot amino acid sequence database which also contained a list of common protein contaminants, plus sequence-reversed decoy entries to establish false discovery rates using the University of Wisconsin’s Mass Spectrometry and Proteomics Facility’s *Mascot* search engine 2.2.07 (Matrix Science) with variable methionine oxidation, variable asparagine and glutamine deamidation, plus fixed cysteine carbamidomethylation. Peptide precursor mass tolerance was set at 15 ppm and fragment mass tolerance at 0.6 Da. Protein annotations, significance of identification and spectral counting based quantification were done using the Scaffold software package (version 4.3.2, Proteome Software Inc., Portland, OR, USA). Protein identifications were accepted if they could be established at greater than 99.0% probability within 1% false discovery rate and contained at least 5 identified peptides. Protein probabilities were assigned by the Protein Prophet algorithm [16]. Proteins that contained similar peptides and could not be differentiated based on MS/MS analysis alone were grouped to satisfy the principles of parsimony.

## 4. Results and Discussion

Orange-colored stains were apparent in the native gels where the reaction with either substrate, i.e., maltose (Figure 1A) or sucrose (Figure 1B), took place. The intensity of the bands could be increased by extending incubation times in the assay reagent. Maltase activity was apparently greater because the develop reagent required less time to be visualized for the staining than for sucrase (Table 1). The locations of the orange-colored stains on the sucrose and maltose gels are above the molecular mass (Mr) marker 460, which is also the case for the two most intense bands of proteins that ran through the Coomassie blue stained gel (intense bands marked with arrows in Figure 1C). Considering this proximity, and that α-glucosidases are the two most abundant proteins in the orange colored bands (see below), we suspect that the bands indicated by the arrows on the Coomassie blue stained gel correspond to SI and MGAM, but additional tests (e.g., Western blots) would be necessary to confirm this. Because electrophoretic mobility of native proteins results from size and charge as well as the overall bulk or cross-sectional area of the protein, Mr markers do not allow the exact determination of the Mr of test proteins [17]. Additionally, the differences in migration over the polyacrylamide gel between the weight marker and the band that contains the α-glucosidases might also be due to the level of glycosylation of these two proteins [4,18]. 

Mass spectrometry results from the excised orange stained bands revealed the presence of 32 proteins (Table 2; all proteins detected are listed in Supplementary Table S1), which was <5% of 650 proteins that were identified in crude bbm without electrophoresis (unpublished data). Of the 32 proteins, 51 ± 2% (S.D.; *n* = 2 mice) of those were hydrolases of which 75 ± 1% were SI and MGAM (Table 2). SI and MGAM overlap in their hydrolysis activity for some substrates, while for others they are unique. For instance, α-1,4 bonds characteristic of the disaccharide maltose are hydrolyzed by both subunits, *N*t and *C*t, of SI and *N*t-MGAM [8,9]. Thus, the orange spot on the gel incubated with sucrose accounts for the activity of the α-1, 2 action of *C*t-SI (Figure 1B) [8,9]. Alternatively, the band that is developed in the gel incubated with maltose accounts for the summed hydrolysis of both enzyme complexes, retrieving the total maltase activity for that given sample (Figure 1A). The fact that we do not see two bands for both complexes is because we maximized the color reaction to develop all the potential glucose formed in the gel. Early in the time course of the color reaction development for glucose, we observed the apparent two incipient bands until they merged (not shown).

Our method description here relied on technical replicates of proteins run on gels (e.g., gels I and II) that were derived from bbm of two mice (two biological replicates). We had similar success applying the method to laboratory rats, chickens, and three species of wild birds (n = 2 individuals/species) [19], so we have become confident in the method’s applicability and replicability. The method can identify the activity of α-glucosidases, but we consider that it can be broadly applied to any enzyme that releases D-glucose or even other products of breakdown that can be identified in gel runs. Furthermore, an indication of the relative ratios of activity can be obtained by comparing the relative staining intensities. After identifying the proteins and assessing their function, their relative abundance relative to other proteins in the bbm components (Table 2) can be semi-quantified using nano LC-MS/MS loading equal amounts of total protein per sample.

## Figures and Tables

**Table 1:** Summary of parameters used with each substrate tested in the zymography.

Electrophoresis		
**Gel**	**I**	**II**
Constituents ^1^	4–12% mini polyacrylamide gel ^1^	
Temperature of the run	room temperature	
Total protein loaded per well	10 µg	
Running buffer	Tris-glycine pH 8.3	
Electric field applied	100 V constant and ~350 mA	
Run time	3 h	
Hydrolytic activity assay		
Substrate solution and concentration	Maltose 56 mM	Sucrose 56 mM
Incubation time	1 h	1 h
Incubation temperature	37 °C	
Assay reagent ^2^ Incubation time (reaction temperature)	1 h (37 °C)	5 h (37 °C)
Rinse the gel with deionized water	3 ×	
Gel storage for further analysis	4 °C	

References: ^1^ Novex^TM^ 4–12% Tris-glycine mini polyacrylamide gel; ^2^ Glucose (GO) Assay Kit (Sigma GAGO-20).

**Table 2:** Hydrolases identified by nano-liquid chromatography coupled to mass spectrometry in the slices cut from gels incubated with maltose and sucrose. Quantitative value and coverage of enzymes in the analyzed sample was determined using Scaffold software package (version 4.3.2, Proteome Software Inc., Portland, OR, USA). The % coverage refers to the percentage of the protein sequence covered by identified peptides. Protein identifications were accepted if they could be established at greater than 99.0% probability with a 1% false discovery rate and containing at least 5 identified peptides.

Enzyme	Uniprot Accesion Number	Molecular Mass (kDa)	Maltose Substrate				Sucrose Substrate			
			Mouse 1		Mouse 2		Mouse 1		Mouse 2	
			Quantitative Value (Normalized Total Spectra)	Coverage %	Quantitative Value (Normalized Total Spectra)	Coverage %	Quantitative Value (Normalized Total Spectra)	Coverage %	Quantitative Value (Normalized Total Spectra)	Coverage %
SI (sucrase-isomaltase)	F8VQM5	209	104	23	42	3	100	35	81	6
MGAM (maltase-glucoamylase)	B5THE2	209	56	15	94	6	83	31	38	4
ANPEP (aminopeptidase-N)	P97449	110	25	11	42	2	49	39	49	5
ENPEP (Glutamyl Aminopeptidase)	P16406	108	9	8	-	-	12	21	-	-
DPP4 (Dipeptidyl peptidase 4)	P28843	87	3	4	-	-	7	16	-	-
MEP1B (Meprin A Subunit Beta)	Q61847	80	3	10	-	-	5	12	-	-
All other non-hydrolase proteins (details in Supplementary Table S1)			194		147		210		211	
Hydrolases as % of total proteins			51%		55%		55%		44%	
α-glucosidases as % of total hydrolases			80%		76%		71%		71%	

## Supplementary Materials

The following are available online at https://www.mdpi.com/2409-9279/3/1/15/s1, Table S1: Proteins identified by nano-liquid chromatography coupled to mass spectrometry in the slices cut from gels incubated with maltose and sucrose.
