# Supplementary material for: A Fast and Accurate Method to Identify and Quantify Enzymes in Brush-Border Membranes: In Situ Hydrolysis Followed by Nano LC-MS/MS
Source: Methods Protoc. 2020 Feb 10;3(1):15. doi: 10.3390/mps3010015 (PMC7189658; doi:10.3390/mps3010015)
Supplement: Supplementary file 1 [file mps-03-00015-s001.pdf]

methods and protocols

MDPI

Protocol

A Fast and Accurate Method to Identify and Quantify Enzymes in Brush-Border Membranes: In Situ Hydrolysis Followed by Nano LC-MS/MS

Antonio Brun [1,2,†], Melisa E. Magallanes [1,†], Carlos Martínez del Rio [3], Gregory A. Barrett-Wilt [4], William H. Karasov [4] and Enrique Caviedes-Vidal [1,5,6,*]

1 Instituto Multidisciplinario de Investigaciones Biológicas de San Luis (IMIBIO-SL), Consejo Nacional de Investigaciones Científicas y Técnicas (CONICET), 5700 San Luis, Argentina; bruntonio@gmail.com (A.B.); melisa.eliana.magallanes@gmail.com (M.E.M.)

2 Facultad de Ciencias de la Salud. Universidad Nacional de San Luis, 5700 San Luis, Argentina

3 Department of Zoology and Physiology, University of Wyoming, Laramie, WY 82071, USA; CmDelRio@uwyo.edu

4 Mass Spectrometry and Proteomics Facility, Biotechnology Center, University of Wisconsin-Madison, WI 53706, USA; barrettwilt@wisc.edu (G.B.-W.); wkarasov@wisc.edu (W.H.K.)

5 Department of Forest and Wildlife Ecology, University of Wisconsin-Madison, Madison, WI 53706, USA

6 Departamento de Biología, Universidad Nacional de San Luis (UNSL), 5700 San Luis, Argentina

† These authors contributed equally to this work

* Correspondence: enrique.caviedes@gmail.com (E.C.-V)

Received: 7 December 2019; Accepted: 3 February 2020; Published: date

Supplementary Table S1. Proteins identified by nano-liquid chromatography coupled to mass spectrometry in the slices cut from gels incubated with maltose and sucrose. Quantitative value and coverage of proteins in the analyzed sample was determined using Scaffold software package (version 4.3.2, Proteome Software Inc., Portland, OR). The % coverage refers to the percentage of the protein sequence covered by identified peptides. Proteins were included if their identity could be established at greater than 99.0% probability with a 1% false discovery rate and containing at least 5 identified peptides.

Identified Proteins = 32	UniProt accession Number	Molecular mass (kDa)	Maltose substrate				Sucrose substrate			
			Mouse 1		Mouse 2		Mouse 1		Mouse 2	
			Quantitative Value*	Coverage %	Quantitative Value*	Coverage %	Quantitative Value*	Coverage %	Quantitative Value*	Coverage %
Sucrase-isomaltase	F8VQM5	209	104	23	42	3	100	35	81	6
Maltase-glucoamylase	B5THE2	209	56	15	94	5	83	31	38	4
Aminopeptidase N	P97449	110	25	12	42	2	49	39	49	5
Actin	P63260	42	30	40	10	4	16	53	38	7
Myosin-14	K3W4R2	229	23	7	-	-	18	18	-	-
Sodium/potassium- transporting ATPase subunit alpha-1	Q8VDN2	113	20	22	21	3	13	22	59	15
Sodium/glucose cotransporter 1	Q8C3K6	73	8	12	-	-	15	25	-	-
Keratin, type I cytoskeletal 19	P19001	45	22	40	-	-	19	61	-	-
ATP synthase subunit alpha	D3Z6F5	55	4	12	73	16	9	25	76	18
Keratin, type II cytoskeletal 8	P11679	55	10	11	-	-	19	43	-	-
Histone H2B type 1-F/J/L	P10853	14	10	30	-	-	18	42	-	-
Voltage-dependent anion- selective channel protein 1	Q60932	32	13	26	10	3	15	49	11	3
Glutamyl aminopeptidase	P16406	108	9	8	-	-	12	21	-	-
ATP synthase subunit beta, mitochondrial	P56480	56	10	23	10	5	16	33	11	5
Fc fragment of IgG-binding protein	E9Q9C6	275	7	5	-	-	6	5	-	-
Plectin	Q9QXS1	534	4	2	-	-	5	3	-	-
Dipeptidyl peptidase 4	P28843	87	3	4	-	-	7	16	-	-
Lactase	F8VPT3	218	1	1	-	-	5	5	-	-
Voltage-dependent anion- selective channel protein 2	G3UX26	30	9	22	-	-	5	34	-	-
Mitochondrial amidoxime reducing component 2	Q922Q1	38	3	12	-	-	3	15	-	-
Amine oxidase [flavin- containing] A	Q64133	60	3	7	-	-	3	11	-	-

Table S1. *Cont.*

Histone H4	P62806	11	5	39	-	-	4	52	-	-
Meprin A subunit beta	Q61847	80	3	10	-	-	5	12	-	-
Villin-1	Q62468	93	3	5	-	-	2	7	-	-
Neutral and basic amino acid transport protein rBAT	Q91WV7	78	2	4	-	-	3	8	-	-
4F2 cell-surface antigen heavy chain	P10852	58	2	7	-	-	4	16	-	-
ATP synthase subunit gamma	Q8C2Q8	30	-	-	21	8	-	-	16	20
Sodium/potassium- transporting ATPase subunit beta-1	P14094	35	2	9	-	-	3	16	-	-
ADP/ATP translocase 2	P51881	33	2	8	-	-	2	18	-	-
Cingulin	D3YUW7	136	1	1	-	-	3	4	-	-
ATP-dependent translocase ABCB1	P21447	141	1	1	-	-	2	5	-	-
Cytochrome b-c1 complex subunit 2, mitochondrial	Q9DB77	48	1	2	-	-	2	14	-	-

Reference. *: normalized total spectra
